# Sequential changes to intraocular lens dislocation caused by vigorous ocular massage: A case report

**DOI:** 10.1097/MD.0000000000020069

**Published:** 2020-05-15

**Authors:** Wei-Shan Tsai, Yuan-Chieh Lee

**Affiliations:** a Department of Ophthalmology, Buddhist Tzu Chi General Hospital; b Department of Ophthalmology and Visual Science; c Institute of Medical Science, Tzu Chi University, Hualien, Taiwan.

**Keywords:** case report, curvilinear tears, intraocular lens dislocation, ocular massage

## Abstract

**Rationale:**

Although intraocular lens (IOL) dislocations have been reported after uneventful cataract surgeries, no sequential changes have ever been demonstrated. Our case showed the sequential changes to IOL dislocation caused by vigorous ocular massage.

**Patient concerns:**

A 42-year-old man complained of blurred vision in the left eye 8 years after uneventful phacoemulsification. The IOL was still well-centered, but curvilinear tears of the anterior and posterior capsule along the optic border of IOL and vitreous herniation were noted. In the following month, the IOL subluxated inferiorly. A careful history taking revealed a recent habit of vigorous ocular massage. The subluxation was stable for 2 years after avoiding ocular massage, but dislocation into vitreous occurred after taking a spring water bath (spa) bath with massage.

**Diagnosis:**

IOL dislocation.

**Interventions:**

Pars plana vitrectomy to remove the dislocated IOL and implantation of a 3-piece IOL into sulcus were performed.

**Outcomes:**

The IOL was well-centered. The visual acuity returned to 20/20.

**Lessons:**

Ocular massage might cause tear of the intact fibrotic capsule and dislocation of IOL. The capsule along the border of the optics might be a weak point against ocular massage.

## 1 Introduction

Intraocular lens (IOL) dislocation is a rare late complication of cataract surgery. The cumulative risk of late posterior chamber IOL dislocation is between 0.1% and 1.7%.^[1]^ Major predisposing factors for in-the-bag dislocation are pseudoexfoliation, retinitis pigmentosa, trauma, the status after vitrectomy, and a long axis, whereas those for out-of-the-bag dislocation are secondary IOL implantation, posterior capsule rupture, and mature cataract.^[2]^ To our knowledge, only 5 cases of rubbing-related IOL dislocation have been reported (3 in-the-bag IOL dislocations and 2 anterior chamber IOL posterior dislocations).^[3,4,5,6]^ Our report is the first to demonstrate the sequential progression to IOL dislocation. Written informed consent for the publication of this case and any additional related information was taken from the patient involved in the study.

## 2 Case report

**Figure 1:**
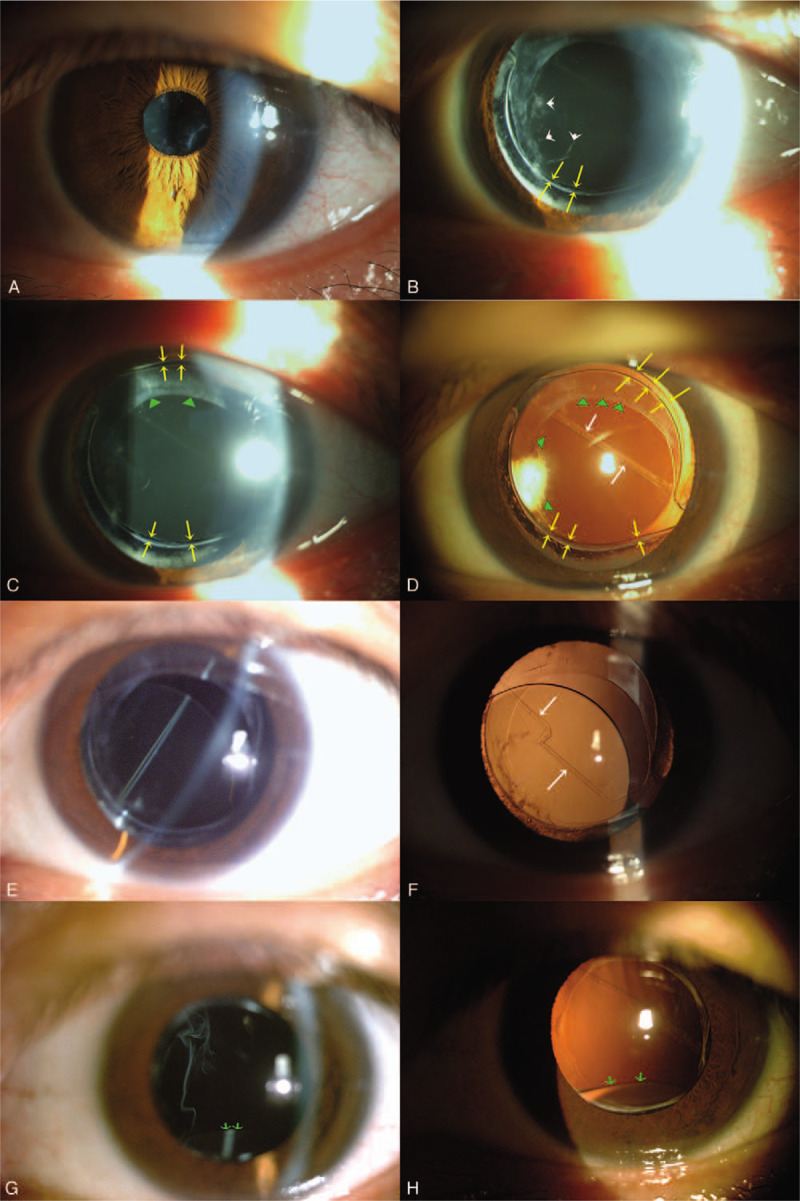
Sequential changes to intraocular lens dislocation. (A) Biomicroscopic examination revealed some feathery whitish material behind the pupil but in front of the IOL. (B) After pupil dilation, vitreous strands (white short arrows) was noted to herniate through the tear (yellow arrows) along the optic border of IOL. (C, D) Two curvilinear tears (yellow arrows) along the superior and inferior optic border of IOL were noted. Retroillumination showed that the posterior capsule was coiled into a visible strand (white long arrows) crossing the optic zone and connecting the superior and inferior haptics; while the anterior capsule between original anterior continuous curvilinear capsulotomy (green arrowheads) and curvilinear tears (yellow arrows) still adhered to the IOL optics. The gap caused by curvilinear tear was clearly visible by retroillumination. (E) In the following month, the patient came back with an inferiorly subluxated IOL after the vigorous ocular massage. (F) Retroillumination showed a loosened coiled strand of the posterior capsule (while long arrows). The curvilinear tear of posterior capsule might extend further to the peripheral haptic area. (G, H) IOL (green arrows) dislocation to inferior vitreous occurred after taking a spa with massage. IOL = intraocular lens, spa = spring water bath.

A 42-year-old high myopic professor visited our clinic with the chief complaint of blurred vision in his left eye for a long time. He had undergone cataract extraction and IOL implantation in his right eye at another hospital before. Best corrected visual acuity (BCVA) was 20/20 in the right eye (OD) and 20/200 in the left eye (OS). Biomicroscopic examination showed pseudophakia (OD) and anterior cortical opacity and nuclear sclerosis (OS). The patient then underwent uneventful phacoemulsification in his left eye with a smooth round continuous curvilinear capsulorrhexis (CCC), intact posterior capsule and in-the-bag IOL implantation (SN60WF, Alcon). The post-operative corrected visual acuity was 20/20. The condition was quite well until 8 years later when he came back with the complaint of mild blurred vision in his left eye. He denied any ocular trauma or any dangerous sports. The corrected visual acuity was still 20/20 in his left eye. Biomicroscopic examination revealed some feathery whitih material behind the pupil but in front of the IOL (Fig. 1A). After pupil dilation, continuous curvilinear tears of the anterior and posterior capsule along the optic border of IOL and vitreous strands herniation were noted (Fig. 1B-D). The IOL was still in its position supported by the fibrotic capsule around the two haptics. Posterior capsule became coiled, crossing the optic zone and connecting the superior and inferior haptics; while the anterior capsule between original CCC and curvilinear tears still adhered to the IOL optics (Fig. 1D). The patient came back with an inferiorly subluxated IOL in 1 month (Fig. 1E, F). The corrected visual acuity decreased to 20/25. A careful history taking revealed the habit of vigorous ocular massage. Because the visual acuity was not so bad and the patient's personal reason, he hesitated about the operation. He was educated to discontinue the habit of ocular massage. The subluxated IOL was stable for 1 and a half years. Unfortunately, a further drop of vision due to IOL dislocation to inferior vitreous occurred after taking a spa with massage (Fig. 1G, H). 25-gauge Pars plana vitrectomy to release the potential vitreous traction, removal of the dislocated IOL and a 3-piece IOL (ZA9003, Abbott) implantation into sulcus with scleral fixation were performed. The visual acuity returned to 20/20 postoperatively. The IOL was well centered and stable in the following 2 years.

## 3 Discussion

Our patient had no risk factors for out-of-the-bag dislocation as reported.^[2]^ The posterior capsule rupture occurred and progressed during the habit of ocular massage and came to a halt after stopping the ocular massage. Therefore, we believe that ocular massage was the culprit of the out-of-the-bag IOL dislocation.

Literature review revealed 5 cases of IOL subluxation or dislocation associated with eye rubbing. In 2001, Yamazaki et al reported the first IOL in-the-bag subluxation into the anterior chamber in a 66-year-old Japanese with atopic dermatitis after forceful eye rubbing, which was treated by IOL removal and contact lens wear.^[3]^ In 2004, Gross et al reported another in-the-bag IOL dislocation from eye rubbing in a 75-year-old female, which was managed by IOL removal in exchange for anterior chamber IOL.^[4]^ In 2005, Poh et al reported a 61-year-old Chinese man with bilateral anterior chamber IOL posterior dislocation into the vitreous cavity after uncontrolled eye rubbing, which was treated by IOL removal via pars plana vitrectomy.^[5]^ In 2007, Gürlü et al reported another anterior chamber IOL posterior dislocation after habitual eye rubbing in a 77-year-old woman.^[6]^ In 2016, Bassily et al reported a 36-year-old woman with atopic eczema suffered from bilateral posterior capsule rupture and IOL dislocation after excessive eye rubbing, which were managed by replacing the dislocated IOL with an iris-claw IOL.^[7]^ In contrast, our patient suffered from posterior capsule rupture and subsequent out-of-the-bag dislocation during the habit of vigorous ocular massage, and received removal of the dislocated IOL and sulcus IOL implantation.

Bassily et al suggested that the mechanism of the posterior capsule tear could have been focal digital pressure on the eye that displaced the IOL vertically, causing the sharp edge of the haptic to rupture the posterior capsule.^[7]^ However, our case beautifully demonstrated that the original posterior capsule tear was along the optic border rather than from the sharp edge of the haptic. Hence, the posterior square edge of optic prevents the formation of posterior capsular opacity, but the square edge might cut the posterior capsule when a forceful pressure is applied on the anterior optic of IOL. The curvilinear tear along the optic border did not only appear on the posterior capsule but also on anterior capsule outside the original CCC. The edge of the optic and a rebound force form the vitreous together might lead to the curvilinear tear of the fibrotic anterior capsule.

Although IOL subluxation or dislocation could be managed with surgical intervention, the best strategy is prevention from happening or progression. We recognized the curvilinear capsule tear when the IOL was still in its original position but failed to prevent its further progression to IOL dislocation in our patient because of the initial lack of thorough history taking. Therefore, both early recognition and a careful history taking of the lifestyle are mandatory.

Our report demonstrated progressive sequential changes to IOL dislocation caused by ocular massage. The initial tears were along the optic border of IOL. Patients should be advised to avoid vigorous ocular massage or excessive eye rubbing, even long after the operation.

## Author contributions

**Conceptualization:** Yuan-Chieh Lee.

**Data curation:** Wei-Shan Tsai, Yuan-Chieh Lee.

**Formal analysis:** Yuan-Chieh Lee.

**Supervision:** Yuan-Chieh Lee.

**Validation:** Yuan-Chieh Lee.

**Writing – original draft:** Wei-Shan Tsai.

**Writing – review and editing:** Yuan-Chieh Lee.
